# ChromPolymerDB: a high-resolution database of single-cell 3D chromatin structures for functional genomics

**DOI:** 10.1093/nar/gkaf1233

**Published:** 2025-11-18

**Authors:** Min Chen, Lin Du, Siyuan Zhao, Bowei Ye, Pourya Delafrouz, Hammad Farooq, Debaleena Chattopadhyay, G Elisabeta Marai, Zhifeng Shao, Jie Liang, Daniel M Czajkowsky, Constantinos Chronis

**Affiliations:** State Key Laboratory of Systems Medicine for Cancer and Bio-ID Center, School of Biomedical Engineering, Shanghai Jiao Tong University, Shanghai 200240, China; Center for Bioinformatics and Quantitative Biology, Richard and Loan Hill Department of Biomedical Engineering, University of Illinois Chicago, Chicago, IL 60607, United States; Electronic Visualization Laboratory, University of Illinois Chicago, Chicago, IL 60607, United States; Center for Bioinformatics and Quantitative Biology, Richard and Loan Hill Department of Biomedical Engineering, University of Illinois Chicago, Chicago, IL 60607, United States; Center for Bioinformatics and Quantitative Biology, Richard and Loan Hill Department of Biomedical Engineering, University of Illinois Chicago, Chicago, IL 60607, United States; Center for Bioinformatics and Quantitative Biology, Richard and Loan Hill Department of Biomedical Engineering, University of Illinois Chicago, Chicago, IL 60607, United States; Electronic Visualization Laboratory, University of Illinois Chicago, Chicago, IL 60607, United States; Electronic Visualization Laboratory, University of Illinois Chicago, Chicago, IL 60607, United States; State Key Laboratory of Systems Medicine for Cancer and Bio-ID Center, School of Biomedical Engineering, Shanghai Jiao Tong University, Shanghai 200240, China; Center for Bioinformatics and Quantitative Biology, Richard and Loan Hill Department of Biomedical Engineering, University of Illinois Chicago, Chicago, IL 60607, United States; State Key Laboratory of Systems Medicine for Cancer and Bio-ID Center, School of Biomedical Engineering, Shanghai Jiao Tong University, Shanghai 200240, China; Center for Bioinformatics and Quantitative Biology, Richard and Loan Hill Department of Biomedical Engineering, University of Illinois Chicago, Chicago, IL 60607, United States; Department of Biochemistry and Molecular Genetics, University of Illinois Chicago, Chicago, IL 60607, United States

## Abstract

The three-dimensional (3D) organization of chromatin plays a critical role in regulating gene expression and genomic processes like DNA replication, repair, and genome stability. Although these processes occur at the individual-cell level, most chromatin structure data are derived from population-averaged assays, such as Hi-C, obscuring the heterogeneity of single-cell conformations. To address this limitation, we developed a polymer physics-based modeling framework, the sequential Bayesian inference framework (sBIF), that deconvolutes bulk Hi-C data to reconstruct single-cell 3D chromatin conformations. To support a broader use of sBIF, we created ChromPolymerDB, a publicly accessible, high-resolution database of single-cell chromatin structures inferred by sBIF. The database contains ∼10^8^ reconstructed 5 kb-resolution single cell structures, spanning over 60,000 genomic loci across 50 human cell types and experimental conditions. ChromPolymerDB features an interactive web interface with tools for 3D structural analysis and multi-omics integration. Users can explore associations between chromatin conformation and gene expression, epigenetic modifications, and regulatory elements. The platform also supports comparative analyses to identify structural changes across cell types, developmental stages, or disease contexts. ChromPolymerDB offers a unique resource for researchers studying the relationship between genome architecture and gene regulation, and for advancing comparative 3D genomics. ChromPolymerDB is available online at https://chrompolymerdb.bme.uic.edu/.

## Graphical Abstract

**Graphical Abstract:**
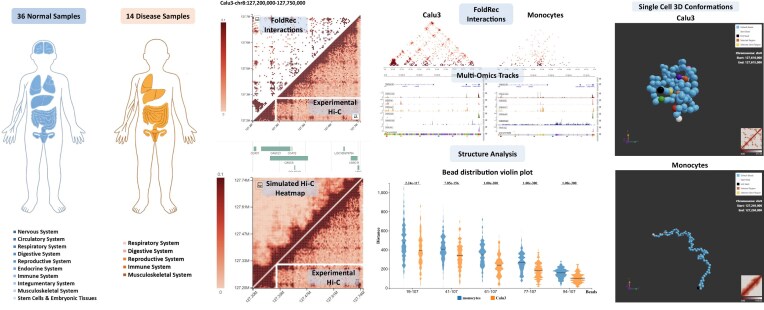

## Introduction

The three-dimensional (3D) organization of chromatin plays a central role in regulating virtually all genomic processes, including gene expression [1–3], DNA replication, and repair [4]. Chromatin architecture underpins the establishment and maintenance of cellular identity and is dynamically remodeled during biological transitions such as differentiation [5–7], development [7–9], and disease progression [10–14]. Advances in chromosome conformation capture methods—particularly Hi-C—have enabled genome-wide mapping of chromatin interactions, revealing that genome function is hierarchically organized across length scales, from inter-chromosomal compartments to topologically associating domains (TADs) and fine-scale chromatin loops [15–25].

Despite these insights, most Hi-C datasets are derived from bulk populations of ∼10^6^ cells [16], yielding ensemble-averaged contact maps that obscure cell-to-cell variability [26]. Although gene regulation and genome function occur at the level of an individual cell, bulk Hi-C does not resolve the precise chromatin architecture within any one cell [27–30]. Imaging studies have confirmed substantial heterogeneity in chromatin conformation across single cells [21, 31], and while single-cell Hi-C methods have emerged to address this limitation [27, 28, 32–34], they remain constrained by extreme data sparsity. The limited number of contacts detected per cell prevents reliable reconstruction of genome-wide 3D structures, particularly at the resolution of TADs and loops [35–38].

To overcome these challenges, we developed the sequential Bayesian inference framework (sBIF), a polymer-physics-based approach for inferring single-cell chromatin conformations from population Hi-C data [39]. sBIF employs a deep-sampling strategy with minimal physical assumptions and no adjustable parameters. It has been validated across species, from *Drosophila* to human [23, 39–41] demonstrating its ability to reproduce bulk Hi-C patterns while capturing chromatin heterogeneity at the single-cell level.

To facilitate the broader application of this approach, we established ChromPolymerDB, a high-resolution, open-access database of single-cell 3D chromatin structures reconstructed using sBIF. The resource includes ∼10^8^ structures at 5 kb resolution, spanning over 60 000 genomic regions across 50 human cell types and experimental conditions. ChromPolymerDB offers a user-friendly web interface equipped with built-in tools for interactive 3D visualization, multi-omics integration, and structural analyses (such as 3D locus distances), within individual samples and across samples, as well as com companion off-line analysis code (such as clustering, TAD radius of gyration, and multi-body contacts). By enabling cross-modality comparisons with transcriptomics, epigenomics, and imaging data, the database facilitates the discovery of structural rewiring events, such as enhancer hub formation or loop remodeling, that can occur during cellular transitions or between disparate cell fates. By facilitating access to and analysis of 3D chromatin architectures, we thus anticipate that ChromPolymerDB will prove to be a useful resource for investigations of chromatin-gene regulation relationships and comparative 3D genomics.

## Materials and methods

### Data collection

To generate ChromPolymerDB, we collected 50 high-quality human Hi-C samples from three major public databases: the 4D Nucleome (4DN) Data Portal [42] (*n* = 19), the ENCODE Portal [43, 44] (*n* = 18), and the Gene Expression Omnibus (GEO) [45] (*n* = 13). To provide data that might be useful to most researchers, the following criteria were used for sample selection: (i) human origin, (ii) sufficient sequencing depth to achieve 5 kb resolution, and (iii) homogeneous cell populations. In some cases, we merged multiple datasets generated under largely similar experimental conditions to achieve higher resolution. Overall, our dataset includes 36 normal cell types (both primary and cultured cells) and 14 disease-associated cell types, covering 10 of the 11 major human physiological systems (except the urinary system).

### Preprocessing Hi-C data for modeling

Following a workflow similar to the original sBIF protocol [39], we applied a four-step pre-processing pipeline to prepare input regions for structural modeling: acquisition of .hic files, TAD boundary identification, TAD-based segmentation of modeling regions, and filtering sparse regions.

1. **Acquisition of .hic files:** Whenever available, hic files aligned to the hg38 reference genome were directly downloaded from the source database. If only hg19-aligned .hic files were provided, we converted them to hg38 using HiCLift [46]. In cases where no .hic files existed, we generated them from raw fastq read files as described in the Supplementary Methods.
2. **TAD boundary calling:** We identified TAD boundaries using OnTAD [47] at a 50 kb resolution with relaxed parameters (penalty = 0, ldiff = 0.25, lsize = 2, minsz = 3 and -maxsz = 80) to ensure broad genome coverage for downstream structure modeling. Level 1 (outermost) TADs were selected as modeling units as they generally encompass complete domain structures.
3. **TAD-based segmentation of modeling regions:** Modeling regions were defined by outermost TAD boundaries. Small TADs (<1Mb) were merged with neighboring domains if the combined size did not exceed 3.5 Mb, avoiding regions that are too small for reliable modeling.
4. **Filtering sparse regions:** Regions with extremely low contact frequency, defined as <1% of bins containing any contacts, were excluded. All contacts within such regions were removed from further analysis to improve modeling accuracy.

### Single-cell 3D structure reconstruction

We used CHROMATIX [48] to identify statistically significant folding reconstitutive (FoldRec) Hi-C interactions at 5 kb resolution. These interactions were used both for structural modeling and for visualization, enabling comparison with experimental Hi-C data (of similar resolution) and supporting downstream functional analysis. To generate sample-specific background models, we applied a fractal Monte Carlo approach to simulate large ensembles of chromatin fibers (500,000 conformations per sample) confined within the nuclear volume. These null ensembles incorporated only polymer physics and nuclear volume exclusion constraints, with nuclear sizes listed for each sample in Supplementary Table [49–74]. Using a Bag of Little Bootstraps resampling approach, we derived null distributions of random chromatin contacts. Experimentally measured population Hi-C contacts were then compared to their corresponding null distributions, and interactions with a *p.adj* < 0.05 were retained as statistically significant FoldRec contacts. Single-cell chromatin structures were reconstructed from these FoldRec interactions using sBIF [39], which applies sequential Bayesian inference. For each dataset, we generated an ensemble of 5000 single-cell chromatin conformations. Within each model, two beads were considered to be in contact if their Euclidean distance was <80 nm (interpreted as a spherical distance threshold), consistent with prior studies [23, 39].

### Database architecture and web implementation

ChromPolyerDB is implemented using a modern architecture. The backend is built with Flask, which provides RESTful APIs and handles core business logic. The frontend, developed in React with the Ant Design component library, delivers a consistent and responsive user interface. Data visualization is powered by D3.js for interactive 2D charts, an embedded IGV.js [75] genome browser provides track-based views of sequencing signals and annotations directly in the app, enabling intuitive exploration of biological datasets, and by Three.js for an in-browser 3D chromosome viewer that supports real-time interaction with structural models. All primary data are stored in a PostgreSQL database, enabling complex relational queries and scalability to large datasets. To improve performance, Redis is used as an in-memory caching layer, minimizing latency for frequently accessed data.

## Results

### Database overview

**Figure 1.**
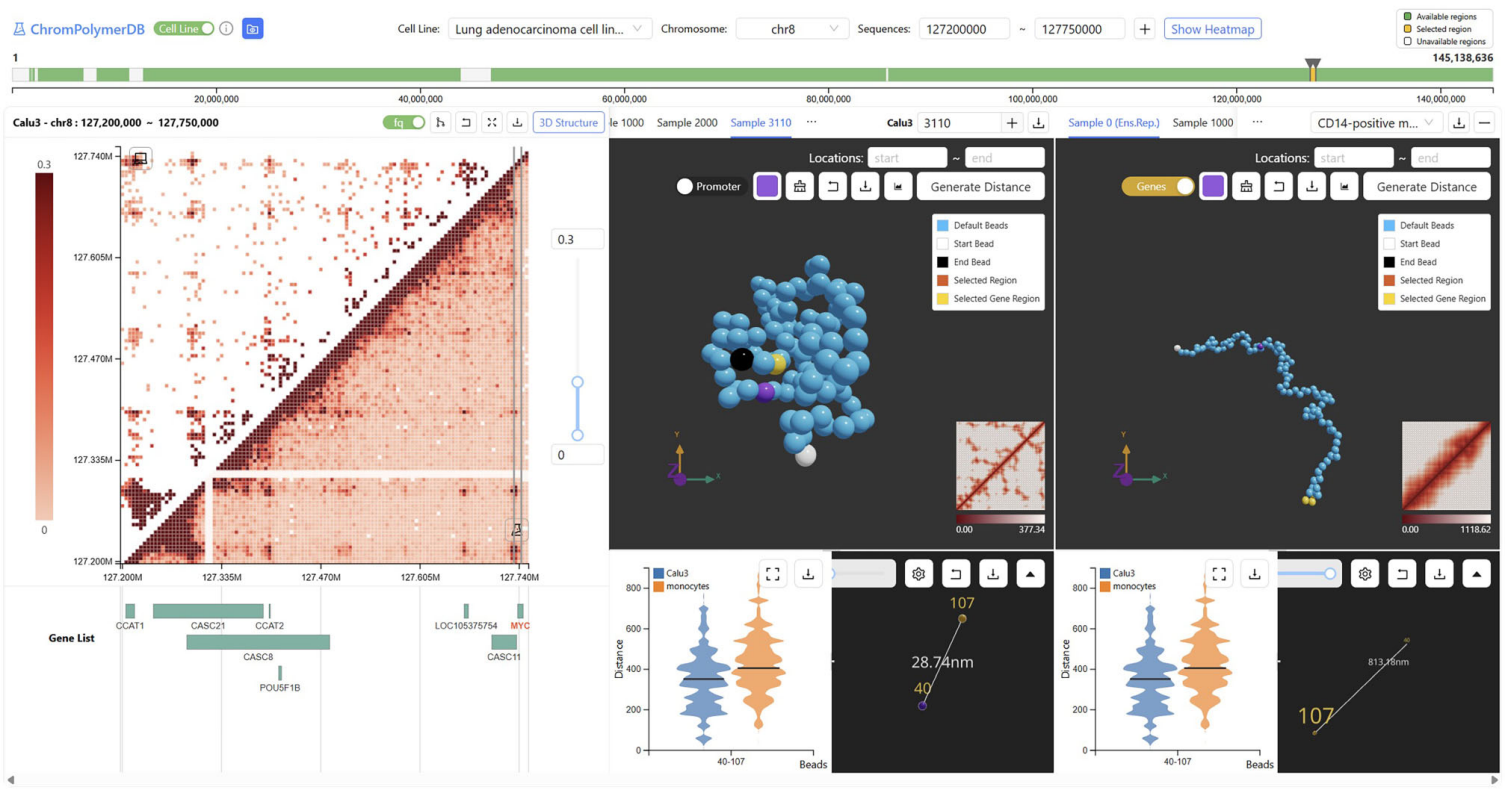
Overview of the ChromPolymerDB database. The example illustrates FoldRec-identified interactions and experimental Hi-C data in Calu3 cells, representative single-cell chromatin conformations from Calu3 and IMR90 cells, and calculated distance distributions between genomic elements of interest.

ChromPolymerDB is a comprehensive, publicly accessible resource that hosts large-scale, high-resolution, single-cell 3D chromatin data, coupled with an interactive web interface for structural analysis and multi-omics integration capabilities (Fig. 1). The database contains ∼10^8^ individual chromatin conformations reconstructed using sBIF, spanning >60 000 genomic regions at 5 kb resolution across 50 human cell types and experimental conditions (Supplementary Table S1). These datasets encompass 36 normal, healthy cell types and 14 disease samples, collectively representing 10 of the 11 major human physiological systems (excluding the urinary system). Beyond data access, ChromPolymerDB offers a suite of analytical tools, including locus-specific 3D visualization, structural measurements, and multi-omics integration, enabling both detailed single-sample interrogations and comparative analysis across cell types and conditions.

### Single-sample insights: chromatin architectures at the *MYC* locus in human lung cancer cells

ChromPolymerDB enables detailed analysis of 3D chromatin organization within individual samples, supporting spatial regulatory investigations. Users can query statistically significant 2D folding interactions (FoldRec, see Materials and methods) and reconstruct complete 3D genome structures.

**Figure 2.**
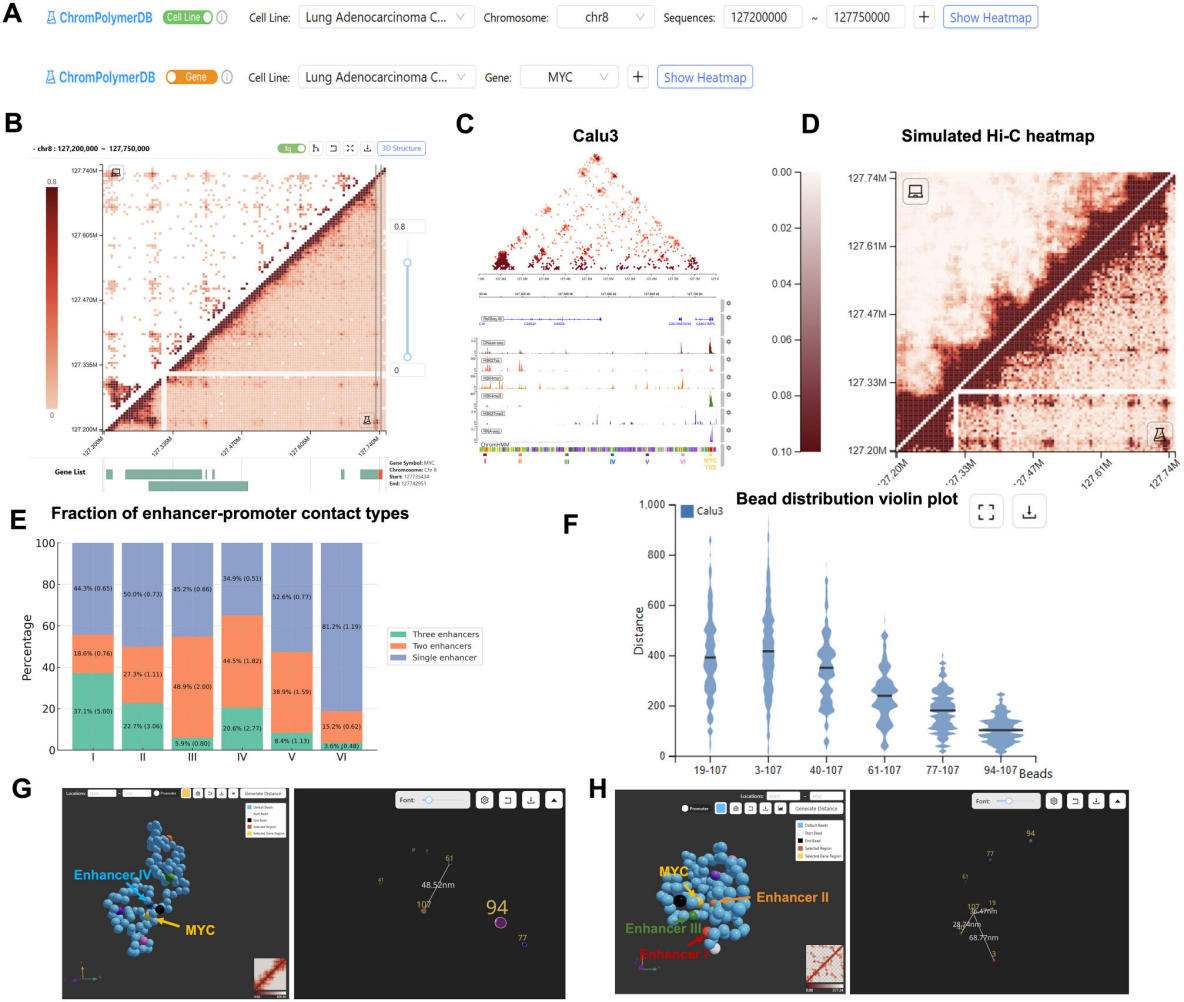
3D chromatin architecture at the *MYC* locus in Calu3 cells. (**A**) Two strategies for querying genomic regions. (**B**) Hi-C heatmap of the *MYC* locus in Calu3 cells (lower triangle: experiment Hi-C; upper triangle: the FoldRec interactions). (**C**) FoldRec interactions overlaid with epigenomic profiles. Six putative enhancers (Enhancers I–VI) are underlined in red (chr8: 127,215,000–127,220,000), orange (chr8: 127,295,000–127,300,000), green (chr8: 127,400,000–127,405,000), blue (chr8: 127,505,000–127,510,000), purple (chr8: 127,585,000–127,590,000), and pink (chr8: 127,670,000–127,675,000). TSS is underlined in yellow (chr8: 127,735,000–127,740,000). (**D**) Comparison between simulated Hi-C heatmap (upper triangle) and experiment Hi-C heatmap (lower triangle). (**E**) Fraction of enhancer–promoter contact types for each putative enhancer, annotated with fold enrichment values. (**F**) Distribution of 3D distances between all putative enhancers and the *MYC* TSS across all generated structures. (**G**) Example single-cell chromatin structure with enhancer IV and the *MYC* TSS highlighted, illustrating close spatial proximity. (**H**) Example single-cell chromatin structure with three putative enhancers (red, orange, and green) and the *MYC* TSS (yellow) highlighted.

As a case study, we analyzed the *MYC* locus (chr8: 127,200,000–127,750,000 bp) in Calu3 lung adenocarcinoma cells, which exhibit high *MYC* expression. *MYC*, a well-known oncogene, is regulated by multiple upstream enhancers [76–78] and plays a central role in tumor initiation and progression [79–81], making it an ideal target for chromatin architecture studies. Before reconstructing of single-cell 3D chromatin structures, we examined 2D Hi-C contact maps and epigenomic profiles to identify putative functional *cis-*regulatory elements. Users can define regions of interest by genomic coordinates or gene name (Fig. 2A). ChromPolymerDB then displays the experiment-derived Hi-C heatmap of that locus (lower triangle) and CHROMATIX-derived FoldRec interactions (upper triangle) (Fig. 2B). In Calu3 cells, the FoldRec interactions reveal contacts between the *MYC* promoter with multiple upstream regions, highlighting the potential regulatory significance of the chromatin organization in this region. Users can further overlay epigenomic tracks directly from the ENCODE Portal or upload their own custom tracks (e.g. histone modifications, transcription factor binding, or chromatin accessibility) for integrated analysis. This analysis enables identification of potential genomic loci of interest, such as regulatory elements, promoters, transcription factor binding sites or other functional regions, thereby facilitating an investigation of how these epigenetic features are related to the chromatin structure. For the *MYC* locus, DNase-seq, H3K27ac, H3K4me1, H3K4me3, and H3K27me3 ChIP-seq profiles, together with ChromHMM annotations, identified six putative regulatory enhancers (Fig. 2D). Several have been experimentally linked to *MYC* regulation in cancers [78, 82], including prostate cancer [83, 84], B-cell malignancies [82], and colorectal cancer [85]. RNA-seq data confirmed *MYC* overexpression in Calu3 cells (Fig. 2C).

Using sBIF, we reconstructed 5000 single-cell chromatin structures for this region, and the most representative single-cell conformations are displayed by default. Users can evaluate the accuracy of the reconstructed models by comparing the aggregate contact map derived from the simulated single-cell structures with the experimental Hi-C contact map (Fig. 2D). The database enables users to switch seamlessly among all available structures for visualization or analysis. Distance measurements can be performed for any selected pair or group loci, such as enhancer–promoter pairs or multi-body contacts. For each selection the 3D distance in the displayed structure and the distribution of pairwise distances across the full set of 5000 models can be calculated. To demonstrate the databases capabilities, we reconstructed single cell chromatin structures of the *MYC* locus (defined as described above) in the Calu3 cells. While bulk Hi-C maps suggest that all putative enhancers can contact the *MYC* TSS, our single-cell reconstructions reveal substantial heterogeneity in these interactions. To quantify this variability, we performed off-line analysis of the bead locations of the 5000 reconstructed single-cell structures and found that 45.5% (2275 in 5000) exhibit at least one enhancer in contact with the *MYC* TSS. Of these 37.5% (1871 in 5000) structures had a single enhancer-promoter contact, 14.8% (336) had two enhancers in proximity, and 3% (68 in 5000) show three enhancers contacting the *MYC* TSS. Thus, ∼18% of enhancer–promoter interactions occur in multibody configurations at the single-cell level, indicating that cooperative enhancer activity is a common feature of *MYC* regulation in these cells.

We further evaluated each putative enhancer’s tendency to participate in multibody interactions with the *MYC* TSS (Fig. 2E). Enhancers I, III, and IV preferentially formed multibody contacts, with Enhancer I strongly enriched for three-enhancer interactions (Fold Enrichment = 5) and Enhancer III and IV favoring two-enhancer contacts (Fold Enrichment = 2 and 1.82). In contrast, Enhancer VI was more often involved in single enhancer–promoter interactions (Fold Enrichment = 1.19). Notably, most three-enhancer multibody interactions were formed by specific enhancer combinations such as (IV, V, and VI) (54.4%), (I, IV, and VI) (20.6%), and (I, II, and III) (8.8%), suggesting that both long-range chromatin folding and local transcription factor binding may underlie cooperative *MYC* regulation.

To illustrate this variability, we highlight two representative structures. In one case (Fig. 2G), only Enhancer IV is in close spatial proximity to the *MYC* promoter, with the remaining five enhancers located further away. In the second example (Fig. 2H), three enhancers (I, II, and III) cluster together with the *MYC* TSS, exemplifying a multibody configuration.

**Figure 3.**
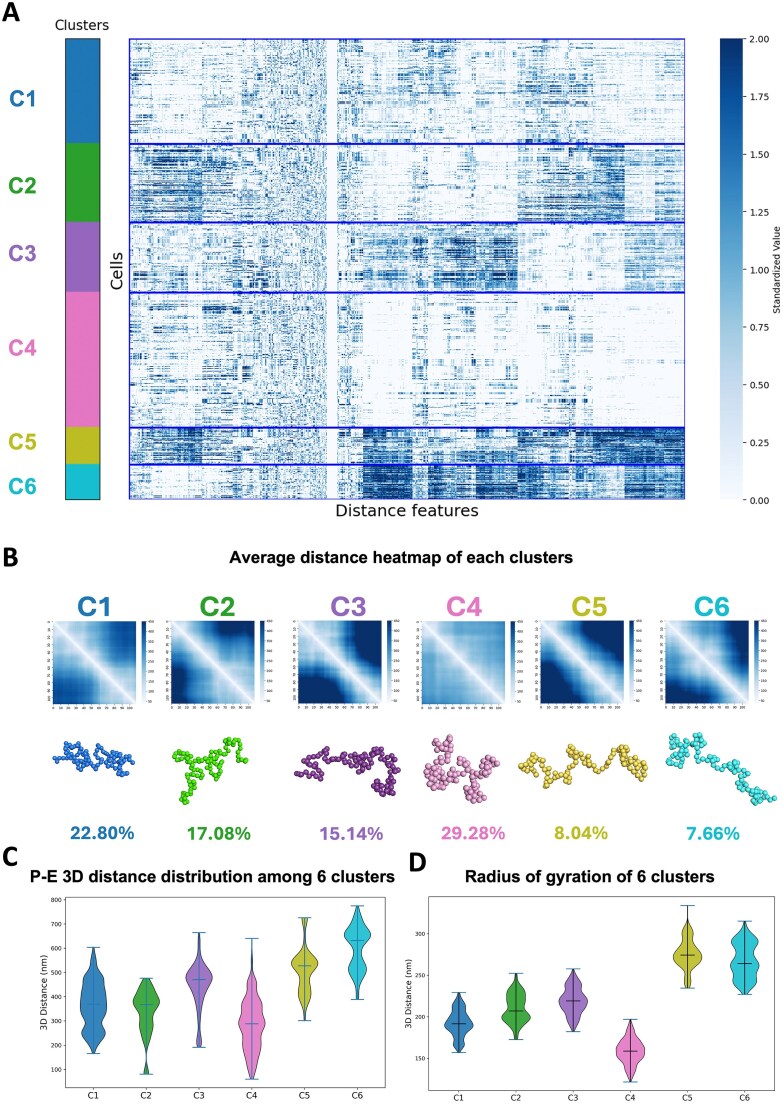
*K*-means clustering of single-cell chromatin structures of the *MYC* locus in Calu3 cells. (**A**) Heatmap of pairwise 3D distance features for all single-cell conformation of the *MYC* locus. (**B**) Average 3D distance heatmaps for each of the five identified subclusters. (**C**) Distribution of 3D distances between putative enhancer III and the *MYC* TSS across the five subclusters. (**D**) Distribution of the radius of gyration for the *MYC* locus in each subcluster.

To further explore this spatial heterogeneity, users can perform ensemble-level clustering of single-cell structures. For the *MYC* locus, *k*-means clustering identified five distinct chromatin subgroups (Fig. 3A and Supplementary Methods), each displaying unique structural patterns (Fig. 3A and B). We also compared features such as the pairwise distance between Enhancer III and the *MYC* promoter (Fig. 3C), as well as the radius of gyration of this region (Fig. 3D), revealing substantial variation across the five subgroups. These structural differences may correspond to distinct regulatory states and could be integrated with other single-cell datasets, such as scRNA-seq or chromatin tracing (see, for example, Supplementary Fig. S1).

### Cross-sample comparison: distinct regulatory mechanisms at the *MYC* locus

ChromPolymerDB also enables direct comparison of chromatin structures across cell types, developmental stages, or disease states, providing a powerful framework for uncovering differences in regulatory mechanisms. The same structural analysis tools described in single-sample insights can be applied to multi-sample datasets, allowing side-by-side evaluation of experimental Hi-C heatmaps, FoldRec interactions, and reconstructed 3D chromatin conformations.

**Figure 4.**
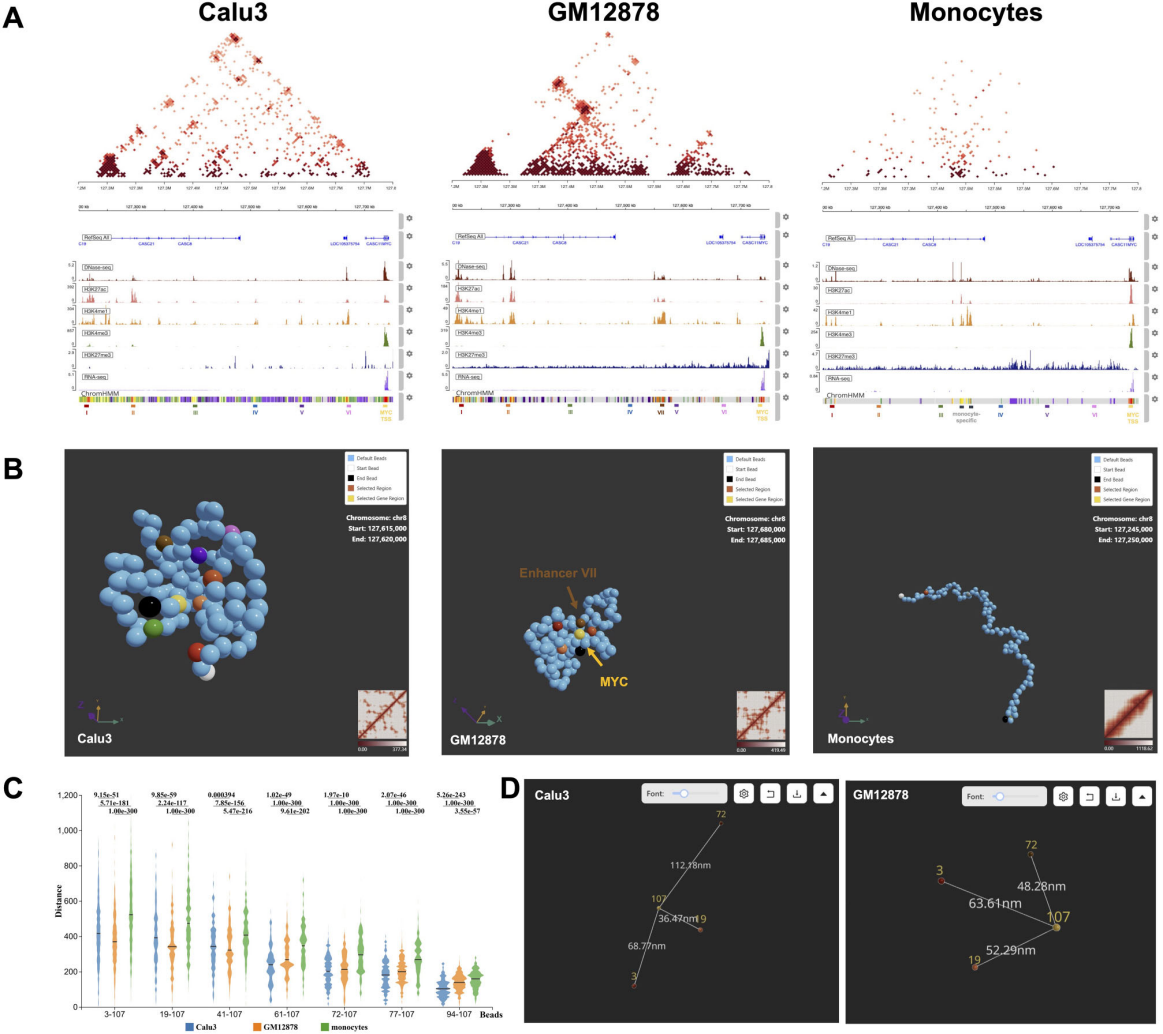
Case study of distinct regulatory mechanisms at the *MYC* Locus across cell types. (**A**) FoldRec interactions overlaid with epigenomic profiles for the *MYC* locus of Calu3 (left), GM12878 (middle), and monocytes (right). In Calu3 cells, six putative enhancers are underlined in red, orange, green, blue, purple, and pink; the *MYC* TSS is underlined in yellow; and the GM12878-specific enhancer is underlined in brown (chr8: 127,560,000–127,565,000). (**B**) Representative single-cell chromatin structures for Calu3 (left), GM12878 (middle), and monocytes (right). (**C**) Distributions of 3D distances between all seven putative enhancers and the *MYC* TSS across all generated structures in Calu3 (left), GM12878 (middle), and monocytes (right). (**D**) Comparison of 3D distances between GM12878 enhancers and the *MYC* TSS in Calu3 (left) and GM12878 (right) structures.

As a demonstration, we compared *MYC* locus regulation in three cell types with contrasting *MYC* expression and epigenetic landscapes: Calu3 lung adenocarcinoma cells (aberrantly high *MYC* expression), GM12878 B-lymphoblastoid cells [15, 42] (high *MYC* expression), and primary CD14^+^ monocytes [43, 44] (negligible *MYC* expression). The observed epigenetic patterns are consistent with RNA-seq profiles displayed in Fig. 4A. Epigenetic analysis (Fig. 4A) revealed that Calu3 and GM12878 share two upstream enhancers (I and II), while Calu3 have four additional enhancers active (III, IV, V, and VI), which are absent in the other two cell types. GM12878 harbors one unique enhancer (Enhancer VII), and monocytes contain two distinct enhancers that lack contact with the *MYC* TSS in the Hi-C data, suggesting that a regulatory relationship is unlikely, based on the available Hi-C data.

We reconstructed 5000 single-cell chromatin structures for all three cell types using sBIF to assess their spatial organization and multibody enhancer–promoter interaction patterns (Fig. 4B and C). In both Calu3 and GM12878, active enhancers were positioned closer to the *MYC* TSS than in monocytes, consistent with the absence of active epigenetic marks and low *MYC* expression in the latter. However, the overall regulatory architectures differed markedly between the three cell types (Fig. 4A).

In Calu3, the most frequent multibody enhancer combinations were enriched, whereas these same configurations were 9.6-fold less frequent in GM12878, likely due to missing enhancer activities in that cell type. GM12878’s active enhancers (I, II, and VII) occasionally colocalized with the *MYC* TSS in the single cell assemblies but did not form stable multibody interactions, indicating a distinct mode of cooperative regulation to Calu3 cells (Fig. 4D). Enhancer–promoter contacts occurred in only ∼10% of GM12878 single-cell structures (446/5000), compared to substantially higher frequencies in Calu3 cells. Among GM12878 contacts, 1.8% involved three enhancer and 10.3% involved two, underscoring the reduced prevalence of multibody configurations in GM12878 than Calu3.

These findings highlight ChromPolymerDB’s capacity to reveal cell type-specific regulatory mechanisms, demonstrating that even with similar *MYC* expression levels, Calu3 and GM12878 employ fundamentally different enhancer repertoires, degrees of multi-assembly, and cell-to-cell variability in chromatin organization.

## Discussion

Recent advances in single-cell technologies have enabled high-resolution investigation of diverse biological modalities including transcription [86, 87], epigenetics modifications [88, 89], and chromatin structures [21, 27, 33]. These approaches have transformed our understanding of cellular heterogeneity, revealing subpopulations and regulatory cell states that remain invisible in bulk analyses. However, generating accurate, high-resolution chromatin structures for individual single cells remains experimentally challenging and computationally intensive, limiting their accessibility to the broader research community.

To address these limitations, we developed sBIF to infer single-cell chromatin architectures from population Hi-C data [39]. While sBIF achieves high structural resolution, its computational cost and technical complexity have restricted widespread adoption. ChromPolymerDB was, therefore, created as a publicly accessible, large-scale resource that delivers, high-resolution single-cell 3D chromatin structures alongside interactive tools for structural visualization, analysis, and multi-omics integration.

Over the past years, several chromatin structure databases have been developed, greatly facilitating research in chromatin organization. For example, databases such as HiChIPdb [90], the 3D Genome Browser [91], 3DIV [92], and LoopCatalog [93] provide cross-species, high-resolution two-dimensional chromatin contact information at the bulk level, offering valuable support and laying the foundation for subsequent advances in chromatin structure research. However, the focus of these databases remains largely on two-dimensional bulk data, with limited coverage of three-dimensional structures and capacity to capture single-cell heterogeneity. In addition, databases such as GSDB [94], Nucleome Browser [95], and 3Disease Browser [96] have provided three-dimensional chromatin structure data with two-dimensional chromatin contact information, representing important progress toward more comprehensive and in-depth insights into chromatin organization. While highly valuable, these resources still have room for improvement in terms of resolution and sample coverage.

In contrast, ChromPolymerDB contains ∼10⁸ single-cell chromatin structures spanning > 60,000 genomic regions at 5 kb resolution, across 50 diverse human cell types and experimental conditions. This scale enables both in-depth analyses within a single cell type and systematic comparisons across multiple cellular contexts.

Utility of our database is illustrated through two case studies at the *MYC* locus. In human lung cancer Calu3 cells, single-cell reconstructions revealed extensive heterogeneity in enhancer–promoter spatial interactions that was obscured in bulk Hi-C data. This heterogeneity likely reflects regulatory variability relevant to *MYC* overexpression and provides hypothesis for targeted functional testing.

Beyond single sample analysis, ChromPolymerDB enables cross-sample comparisons to identify cell type-specific differences in regulatory architecture. Comparing *MYC* locus regulation in Calu3, GM12878, and primary CD14^+^ monocytes revealed marked contrast in enhancer usage, multibody interaction frequency, and spatial variability. These newly discovered differences could not be fully resolved from bulk Hi-C or epigenetic profiles alone. These findings underscore the critical role of single-cell structural data in uncovering the regulatory logic underlying gene expression.

By integrating such structural information with other genomic and epigenomic datasets, ChromPolymerDB will serve as a valuable platform that supports hypothesis generation, comparative 3D genomics, and mechanistic studies of gene regulation. Looking forward, we plan to expand the database to include additional species, incorporate new high-quality Hi-C datasets, and implement advanced structure analysis algorithms and more intuitive user tools. Integration with other single-cell omics modalities, (e.g. scRNA-seq and single-cell epigenomics) will further enable multi-modal analyses, offering a more comprehensive view of genome organization and regulation.

We anticipate that ChromPolymerDB will serve as a key community resource that can bridge the gap between raw chromatin conformation data and biological insight, thereby accelerating research in genome biology, regulatory genomics, and disease-associated chromatin remodeling.

## Supplementary Material

gkaf1233_Supplemental_Files

## Data Availability

All the data are available from the URL: https://chrompolymerdb.bme.uic.edu/.
